# Status Epilepticus after Prolonged Umbilical Cord Occlusion Is Associated with Greater Neural Injury Fetal Sheep at Term-Equivalent

**DOI:** 10.1371/journal.pone.0096530

**Published:** 2014-05-05

**Authors:** Paul P. Drury, Joanne O. Davidson, Lotte G. van den Heuij, Guido Wassink, Eleanor R. Gunn, Lindsea C. Booth, Laura Bennet, Alistair J. Gunn

**Affiliations:** 1 Fetal Physiology and Neuroscience Group, Department of Physiology, The University of Auckland, Auckland, New Zealand; 2 Howard Florey Institute, University of Melbourne, Melbourne, Victoria, Australia; 3 Starship Children’s Hospital, Auckland, New Zealand; Hôpital Robert Debré, France

## Abstract

The majority of pre-clinical studies of hypoxic-ischemic encephalopathy at term-equivalent have focused on either relatively mild insults, or on functional paradigms of cerebral ischemia or hypoxia-ischemia/hypotension. There is surprisingly little information on the responses to single, severe ‘physiological’ insults. In this study we examined the evolution and pattern of neural injury after prolonged umbilical cord occlusion (UCO). 36 chronically instrumented fetal sheep at 125–129 days gestational age (term = 147 days) were subjected to either UCO until mean arterial pressure was < = 8 mmHg (n = 29), or sham occlusion (n = 7). Surviving fetuses were killed after 72 hours for histopathologic assessment with acid-fuchsin thionine. After UCO, 11 fetuses died with intractable hypotension and 5 ewes entered labor and were euthanized. The remaining 13 fetuses showed marked EEG suppression followed by evolving seizures starting at 5.8 (6.8) hours (median (interquartile range)). 6 of 13 developed status epilepticus, which was associated with a transient secondary increase in cortical impedance (a measure of cytotoxic edema, p<0.05). All fetuses showed moderate to severe neuronal loss in the hippocampus and the basal ganglia but mild cortical cell loss (p<0.05 vs sham occlusion). Status epilepticus was associated with more severe terminal hypotension (p<0.05) and subsequently, greater neuronal loss (p<0.05). In conclusion, profound UCO in term-equivalent fetal sheep was associated with delayed seizures, secondary cytotoxic edema, and subcortical injury, consistent with the predominant pattern after peripartum sentinel events at term. It is unclear whether status epilepticus exacerbated cortical injury or was simply a reflection of a longer duration of asphyxia.

## Introduction

Moderate to severe hypoxic-ischemic encephalopathy (HIE) occurs in 1–4 per 1000 live births at term in developed nations [1]. Injury to the basal ganglia, thalamus and white matter is a common pattern of injury, and is highly associated with perinatal sentinel events and subsequent risk of cerebral palsy [2], [3], [4], [5]. Predominant cortical injury in a watershed distribution is also common, but is more typically associated with prolonged partial hypoxia [2], [5]. Most of these cases are associated with acute events around the time of birth [6], [7].

The central finding from pre-clinical studies is that cell death can evolve for many hours after surprisingly severe insults, providing a window of opportunity for intervention [8], [9]. Most of our knowledge of the timing and nature of the evolving electrophysiological changes and patterns of neuronal death in neonatal HIE has been derived from functional models of hypoxia-ischemia (HI), that use a combination of hypoxia with hypotension or ischemia [10], or carotid artery occlusion to produce forebrain ischemia [11], [12], [13]. There is surprisingly limited information on the evolution of injury after severe ‘physiological’ insults such as umbilical cord occlusion (UCO), that involve profound hypoxia with mixed respiratory/metabolic acidosis at term. Most previous studies of UCO in term-equivalent fetal sheep have examined either relatively short insults, which are associated with selective hippocampal injury, without significant seizure activity [14], or repeated or partial UCO [15], [16].

In the present study we examined the hypothesis that prolonged UCO in chronically instrumented un-anesthetized 0.85 gestation fetal sheep, continued until profound hypotension developed (mean arterial blood pressure ≤8 mmHg), would be associated with evolving neural injury as shown by delayed seizures and cytotoxic edema. We tested the secondary hypothesis that periods of continuous seizures (i.e. status epilepticus) would be associated with greater neural injury. At 0.85 gestation brain maturation of the fetal sheep is broadly equivalent to that of the full-term human infant [17], [18].

## Methods

All procedures were approved by the Animal Ethics Committee of the University of Auckland following the New Zealand Animal Welfare Act, and the Code of Ethical Conduct for animals in research established by the Ministry of Primary Industries, Government of New Zealand. 36 singleton Romney/Suffolk fetal sheep were operated on at 121–125 d gestational age (term = 147 days). Food, but not water was withdrawn 18 h before surgery. Ewes were given 5 ml of Streptocin (procaine penicillin (250,000 IU/ml) and dihydrostreptomycin (250 mg/ml), Stockguard Labs Ltd, Hamilton, N.Z.) i.m. 30 min before the start of surgery. Maternal weight was recorded to calculate drug doses. Anesthesia was induced by i.v. injection of propofol (5 mg/kg; AstraZeneca Limited, Auckland, New Zealand), and general anesthesia maintained using 2–3% isoflurane (Medsource, Ashburton, New Zealand) in O_2_. A 20-g i.v. catheter was placed in a maternal front leg vein and the ewes were placed on a constant infusion saline drip to maintain maternal fluid balance. Ewes were ventilated if necessary and the depth of anesthesia, maternal heart rate and respiration were constantly monitored by trained anesthetic staff.

All surgical procedures were performed using sterile techniques [19], [20]. The uterus and either the top or bottom half of the fetus were exteriorized through a maternal midline abdominal incision. Catheters were placed in the left fetal femoral artery and vein, right brachial artery and vein, and the amniotic sac. An ultrasonic blood flow probe (size 3S; Transonic Systems Inc., Ithaca, NY, USA) was placed around the left carotid artery to measure carotid blood flow (CaBF) as an index of global cephalic blood flow [14], [21], [22], [23], [24], and another (size 2R) placed around the right femoral artery to measure femoral blood flow (FBF). Two pairs of electroencephalogram (EEG) electrodes (AS633-5SSF, Cooner Wire Co., Chatsworth, CA, USA) were placed through burr holes on the dura over the parasagittal parietal cortex (10 mm and 20 mm anterior to bregma and 10 mm lateral) and secured with cyanoacrylate glue. To measure cortical impedance a pair of electrodes was placed over the dura, 5 mm lateral to the EEG electrodes. A reference electrode was sewn over the occiput. A thermocouple (Mallinckrodt Mon-a-therm incutemp, Tyco Healthcare, Auckland, New Zealand) was placed 30 mm anterior to bregma over the dura and secured with cyanoacrylate glue. A pair of electrodes was sewn over the fetal chest to measure the fetal electrocardiogram (ECG). An 18–20 mm diameter inflatable silicone occluder was placed around the umbilical cord of all fetuses (In Vivo Metric, Healdsburg, CA, USA). All fetal leads were exteriorized through the maternal flank and a maternal long saphenous vein was catheterized to provide access for post-operative care and euthanasia. 80 mg gentamicin (Rousell, Auckland, New Zealand) was administered into the amniotic sac before closure of the uterus.

Post-operatively all sheep were housed in separate metabolic cages with access to water and food *ad libitum*, together in a temperature-controlled room (16±1°C, humidity 50±10%) with a 12 h light/dark cycle. A period of 4 days post-operative recovery was allowed before experiments commenced, during which time antibiotics were administered to the ewe daily for four days i.v. (600 mg benzylpenicillin sodium; Novartis Ltd, Auckland, New Zealand, and 80 mg gentamicin). Fetal catheters were maintained patent by continuous infusion of heparinized saline (20 U/ml at 0.2 ml/h) and the maternal catheter maintained by daily flushing.

### Experimental Procedures

#### Recordings

Fetal mean arterial blood pressure (MAP), corrected for maternal movement by subtraction of amniotic fluid pressure (Novatrans II, MX860; Medex Inc., Hilliard, OH, USA) [25], CaBF, FBF, ECG, EEG, extra-dural temperature, and impedance were recorded continuously. The blood pressure signal was collected at 64 Hz and low pass filtered at 30 Hz. Carotid vascular conductance (CVC) and femoral vascular conductance (FVC) were calculated as blood flow/(MAP-venous pressure). The EEG signal was high-pass filtered at 1.6 Hz and low-pass filtered at 50 Hz, then stored for offline analysis at a sampling rate of 256 Hz. Cerebral impedance was calculated as previously described [26]. The impedance of a tissue rises concomitantly as cells depolarize and fluid shifts from the extracellular to intracellular space, and thus impedance is a measure of cytotoxic edema. Data were collected by computer and stored to disk for off-line analysis.

#### Experimental protocol

Experiments were conducted at 125–129 d (0.85) gestation. Fetuses were randomly assigned to UCO (n = 29) or sham occlusion (n = 7). Fetal asphyxia was induced between 0900 and 1000 h by rapid inflation of the umbilical cord occluder with sterile saline of a defined volume known to completely inflate the occluder and totally compress the umbilical cord, as determined in pilot experiments with a Transonic flow probe placed around an umbilical vein [23]. Successful occlusion was confirmed by observation of a rapid onset of bradycardia with a rise in MAP, and by pH and blood gas measurements. Occlusion was terminated at a target MAP of 8 mmHg. The duration/depth of occlusion was chosen to represent an acute, severe, near-terminal insult, as determined in previous studies [14], [19], [20], [27]. After release of the umbilical cord occluder fetuses were allowed to auto-resuscitate. If fetal heart rate (FHR) was not above 100 bpm within 1 min of occlusion release then 0.1–0.3 ml/kg of 1/10000 adrenaline (Hospira NZ Limited, Auckland, New Zealand) was administered via the brachial vein, followed by 2 ml of sterile saline to clear the infusion line. This regime was repeated twice if no response was observed within 30 s, followed by dose escalations up to 1 ml/kg. If no response was observed by 10 min then the ewe was euthanized by an intravenous overdose of pentobarbitone sodium (9 g) to the ewe (Pentobarb 300; Chemstock International, Christchurch, New Zealand). Surviving fetuses were allowed to recover for 72 h before post-mortem.

Fetal arterial blood was taken at 15 min prior to asphyxia, at 2 and 12 min during asphyxia, and at +30 min, +1 h, +2 h, +3 h, +4 h, +5 h, +6 h, +24 h, +48 h, and +72 h for pH and blood gas determination (Ciba-Corning Diagnostics 845 blood gas analyzer and co-oximeter, MA., USA) and for glucose and lactate measurements (YSI model 2300, Yellow Springs, Ohio, USA). At the end of the experiment ewes and fetuses were killed as described above.

#### Tissue preparation

Fetal brains were gravity perfusion-fixed in situ with 0.9% saline solution (500 ml) then 10% phosphate-buffered formalin (500 ml) from a height of 1 m, then removed and fixed for a further three days before processing and paraffin embedding [28]. Coronal sections (10 µm) were taken for histological analysis.

#### Histology

For assessment of neuronal loss, brain sections were stained with acid fuchsin-thionin. Prior to staining slides were deparaffinized in xylene (2×15 min), rehydrated in a series of ethanol steps (100%, 95%, 70% for five minutes each), and then washed in PBS for 2 min. Sections were then labeled with thionine acetate (Sigma-Aldrich Pty. Ltd.) for 8 min, dipped in distilled water to wash, then acid fuchsin for 35 s, followed by a quick wash in distilled water. The sections were then dehydrated (dipped 2×95% ethanol, 1×100% ethanol, 2 min in 100% ethanol, xylene 2×15 min) and mounted.

Brain regions of the forebrain used for analysis included the mid-striatum (comprising the caudate nucleus and putamen), the parasagittal cortex and lateral cortex, which were assessed on sections taken 26 mm anterior to stereotaxic zero, according to the stereological atlas of the fetal sheep [29]. The cornu ammonis (CA) of the dorsal horn of the anterior hippocampus (divided into CA1/2, CA3, CA4, and dentate gyrus (DG)) were assessed on sections taken 17 mm anterior to stereotaxic zero.

Neuronal loss was scored on acid fuchsin-thionine stained sections by light microscopy at ×20 or ×40 magnification on a Nikon 80i microscope with a motorized stage and Stereo investigator software V.8 (Microbrightfield Inc; Williston, VT, USA) using seven fields in the striatum (four in caudate nucleus, three in putamen), and one field in each of the hippocampal divisions, lateral cortex, parasagittal cortex, and medial nucleus of the thalamus. The proportion of neurons showing ischemic cell change as shown by nuclear condensation and acid fuchsin (pink) staining of the cytoplasm in each brain region was scored on a six-point scale as follows: 0 =  no dead neurons; 5 = >0% to 10%; 30 = >10% to 50%; 70 = >50% to 90%; 95 = 90% to <100%; and 100 = 100% dead neurons [11]. For each animal, average scores from two sections across both hemispheres were calculated for each region.

#### Data analysis

Off-line analysis of the physiological data was performed using customized Labview programs. Data were analyzed by an investigator blinded to experimental group using SPSS for windows (SPSS 20.0, Chicago, Il, USA). Between group comparisons of serial data were performed by analysis of variance (ANOVA), with time treated as a repeated measure. When statistical significance was found one-way ANOVA was used to compare selected time points. Non-parametric tests were used for non-normal data. For presentation of neurophysiology and histopathology we compared fetuses that developed status epilepticus during recovery (UCO+SE, n = 6) and those that did not (UCO−SE, n = 7). Status epilepticus was identified on the 1 minute EEG record as either continuous seizure activity for >30 min, or a 3 h period with ≥50% seizure activity [30], [31]. Statistical significance was accepted when *P*<0.05. Data are mean±SEM for parametric data and median (interquartile range) for non-parametric data.

## Results

### Baseline and Umbilical Cord Occlusion

**Figure 1:**
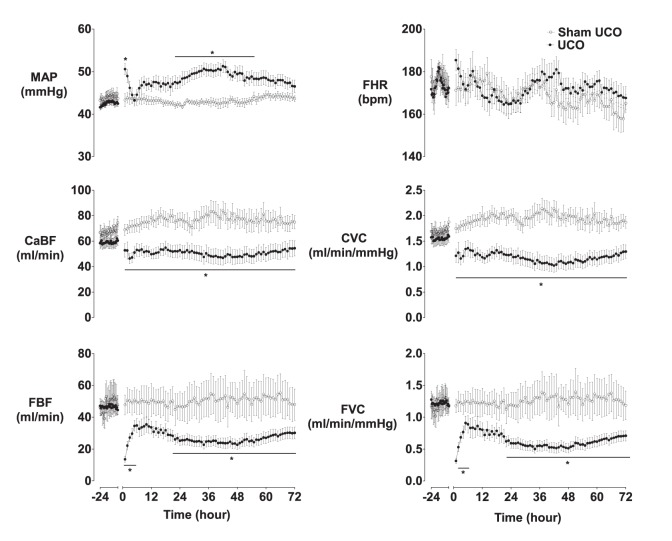
Mean arterial blood pressure (MAP), fetal heart rate (FHR), carotid artery blood flow (CaBF), carotid artery vascular conductance (CVC), femoral artery blood flow (FBF), and femoral artery vascular conductance (FVC) during 24 h baseline and for 72 h recovery after UCO. Occlusion data omitted. *p<0.05 compared to sham occlusion. Data are mean±SEM.

All fetuses had baseline blood gases, acid-base status and glucose-lactate values in the normal range by our laboratory standards. UCO was associated with marked fetal hypoxemia and acidemia (Table 1), increased blood hemoglobin (at 12 min of UCO, p<0.05), sustained bradycardia and peripheral vasoconstriction, with initial hypertension followed by profound hypotension and cerebral hypoperfusion, suppression of the EEG and a delayed rise in cortical impedance (Figure 1). On average fetuses reached the target nadir MAP after 15.9±0.4 min of UCO. The fetuses that later developed status epilepticus (UCO+SE) had a longer mean UCO (17.1±0.5 *vs.* 14.6±0.3 min, p<0.005), and a lower MAP in the final minute of UCO (9.1±0.3 *vs.* 12.7±1.1 mmHg, p<0.05), than fetuses that showed only discrete seizures (UCO−SE). Three fetuses died at the end of UCO from intractable hypotension and 8 fetuses died during the recovery phase. 5 ewes entered labor as shown by regular increases in amniotic pressure occurring at least 3 times per 10 min, and were euthanized. 2 of these 5 fetuses developed status epilepticus. 13 fetuses survived until post-mortem at 72 hours.

**Table 1:** pH, blood gases, glucose and lactate values at baseline, during and after umbilical cord occlusion.

		Baseline	2 min	12 min	+30 min	+1 h	+2 h	+3 h	+4 h	+6 h	+24 h	+48 h	+72 h
**pH**	S	7.39±0.01	7.38±0.01	7.39±0.01	7.39±0.01	7.39±0.01	7.38±0.01	7.39±0.01	7.38±0.01	7.39±0.01	7.38±0.01	7.37±0.00	7.37±0.02
	O	7.38±0.01	7.28±0.01§	6.92±0.01§	7.20±0.01§	7.24±0.02§	7.29±0.02*	7.33±0.02	7.33±0.03	7.34±0.03	7.40±0.01	7.40±0.01§	7.39±0.01
**pCO_2_**	S	53.2±1.6	47.0±1.7	52.8±1.7	48.2±1.8	53.1±1.6	48.6±2.6	52.7±1.7	47.4±1.8	52.9±1.8	52.2±1.5	53.0±2.4	53.4±1.1
(mmHg)	O	52.8±1.4	63.0±1.9§	136.2±3.5§	50.2±1.3	54.0±1.2	48.0±1.0	51.7±1.2	47.8±1.0	51.9±1.5	49.9±1.1	49.8±1.4	51.6±1.2
**pO_2_**	S	23.1±0.9	23.4±1.3	23.6±1.0	23.4±1.5	22.9±0.8	23.7±1.3	23.0±1.3	22.7±1.2	22.1±1.4	23.8±1.1	22.2±0.8	21.6±0.7
(mmHg)	O	22.4±0.9	6.7±0.6§	5.0±0.5§	28.0±1.1*	25.3±1.2	24.1±1.3	22.2±1.2	22.1±1.1	21.1±1.3	21.5±1.1	25.6±1.1	26.2±1.1*
**Hb**	S	10.6±0.3	9.7±0.1	10.4±0.3	10.1±0.1	10.4±0.3	9.7±0.2	10.4±0.3	9.6±0.3	10.2±0.3	9.7±0.3	9.8±0.5	10.2±0.3
(g.dL^−1^)	O	10.4±0.4	10.7±0.3	11.6±0.3*	10.7±0.3	10.9±0.3	10.0±0.3	10.3±0.4	9.8±0.3	10.2±0.4	10.3±0.4	10.3±0.4	9.9±0.5
**Hct**	S	31.2±0.8	28.3±0.4	31.0±0.8	29.3±0.3	30.5±0.9	28.5±0.7	30.5±1.0	28.2±0.7	29.8±0.8	28.5±0.8	28.8±1.7	30.2±1.0
	O	30.6±1.1	31.5±1.0	33.9±1.0	31.4±0.9	32.0±1.0	29.4±1.0	30.4±1.1	28.9±0.9	30.0±1.0	30.5±1.2	30.2±1.2	29.3±1.4
**O_2_ct**	S	4.4±0.3	4.0±0.2	4.4±0.2	4.1±0.3	4.2±0.2	4.1±0.2	4.3±0.3	3.8±0.2	4.2±0.3	4.1±0.2	3.8±0.2	3.9±0.3
(mmol.L^−1^)	O	4.1±0.2	0.5±0.0§	0.5±0.0§	4.2±0.2	4.1±0.2	3.6±0.2	3.7±0.3	3.5±0.2	3.5±0.3	3.7±0.2	4.4±0.2	4.3±0.2
**HCO_3_^−^**	S	29.2±1.0	25.8±1.0	29.2±0.9	26.9±1.2	29.1±0.8	26.2±1.2	28.7±1.1	26.0±1.1	29.2±1.1	28.2±1.0	27.9±1.1	28.2±1.2
(mmol.L^−1^)	O	28.5±0.5	24.5±0.5	17.5±1.5§	17.2±0.3§	19.7±0.5§	20.6±0.8§	24.4±1.1*	23.5±1.4	25.4±1.6	28.5±1.0	28.9±0.6	28.7±0.4
**BE**	S	5.5±1.1	1.9±1.1	5.5±1.0	3.0±1.4	5.3±0.8	2.3±1.4	5.0±1.2	2.1±1.3	5.5±1.2	4.3±1.1	4.0±1.3	4.3±1.7
(mmol.L^−1^)	O	4.5±0.5	0.9±0.5	−8.4±0.4§	−8.8±0.4§	−5.6±0.6§	−4.2±1.0§	0.0±1.4	−1.0±1.8	1.2±1.8	4.8±1.1	5.0±0.5	5.0±0.3
**Lactate**	S	1.1±0.1	1.0±0.1	1.1±0.1	1.1±0.1	1.2±0.1	1.1±0.1	1.3±0.2	1.2±0.1	1.2±0.2	1.2±0.1	1.3±0.1	1.1±0.1
(mmol.L^−1^)	O	1.1±0.1	1.6±0.1§	6.0±0.3§	5.8±0.2§	6.5±0.3§	5.6±0.5§	5.3±0.6§	5.3±0.9*	5.8±1.3*	3.2±0.8	1.3±0.1	1.1±0.1
**Glucose**	S	1.0±0.1	0.8±0.1	0.9±0.1	0.9±0.0	1.0±0.1	0.9±0.0	1.0±0.1	0.9±0.1	0.9±0.1	0.8±0.1	0.8±0.1	0.8±0.1
(mmol.L^−1^)	O	0.8±0.1	0.5±0.0§	1.0±0.1	1.7±0.2*	1.6±0.1*	1.2±0.1*	1.3±0.1*	1.2±0.1*	1.5±0.1§	1.5±0.1§	1.4±0.1*	1.0±0.1

S: sham occlusion. O: umbilical cord occlusion. Hb: haemoglobin concentration; Hct: hematocrit; O_2_ct: oxygen concentration; HCO_3_^−^: bicarbonate; BE: base excess.

*p<0.05;

§ p<0.005 compared to sham occlusion group. Data are mean±SEM.

7/11 fetuses that died after UCO required adrenaline after UCO termination, compared with 2/7 in the UCO−SE group and 4/6 in the UCO+SE group (N.S.). There was no significant difference in the amount of adrenaline received between groups (0 (0.15) ml, 0.4 (0.65) ml, and 0.4 (5.5) ml respectively, N.S.).

### Recovery Phase from UCO

Acidemia resolved after UCO by +3 h (Table 1). Plasma lactate levels were elevated up to +24 h after UCO (p<0.05 *vs.* sham occlusion), and glucose levels were elevated for 48 h (p<0.05). Hemoglobin and hematocrit were not significantly different between groups.

FHR was not significantly different between groups in the recovery period. In contrast, MAP was significantly higher after UCO at +1 h and then from +22–55 h (Figure 1, p<0.05 *vs.* sham occlusion). This was associated with reduced FBF and FVC after UCO from +1–4 h and then +21–72 h (p<0.05 vs. sham occlusion). CaBF and CVC were significantly reduced in the UCO group throughout the recovery period (p<0.05).

**Figure 2:**
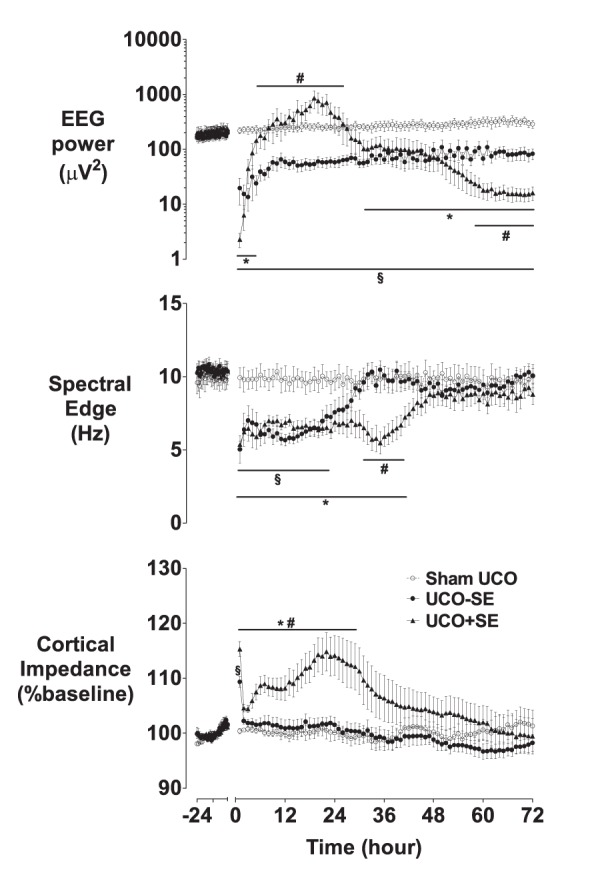
EEG power, spectral edge, and cortical impedance, during 24 h baseline and for 72 h recovery after UCO. Occlusion data omitted. *p<0.05 UCO with status epilepticus vs. sham UCO; #p<0.05 UCO with status epilepticus vs. UCO without status epilepticus; §p<0.05 UCO without status epilepticus vs. sham UCO. Data are mean±SEM.

**Figure 3:**
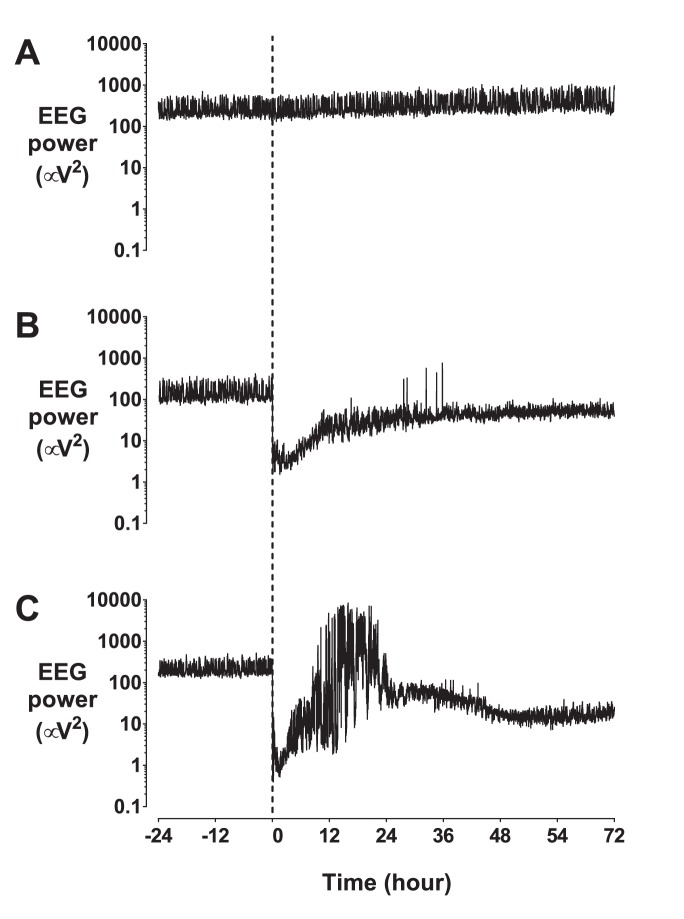
Representative examples of EEG recovery. A: sham occlusion; B: UCO without status epilepticus in recovery; C: UCO with status epilepticus in recovery. Data are 1 minute mean. Umbilical cord occlusion or sham occlusion was initiated at time = 0 h (shown by the vertical dotted line).

**Figure 4:**
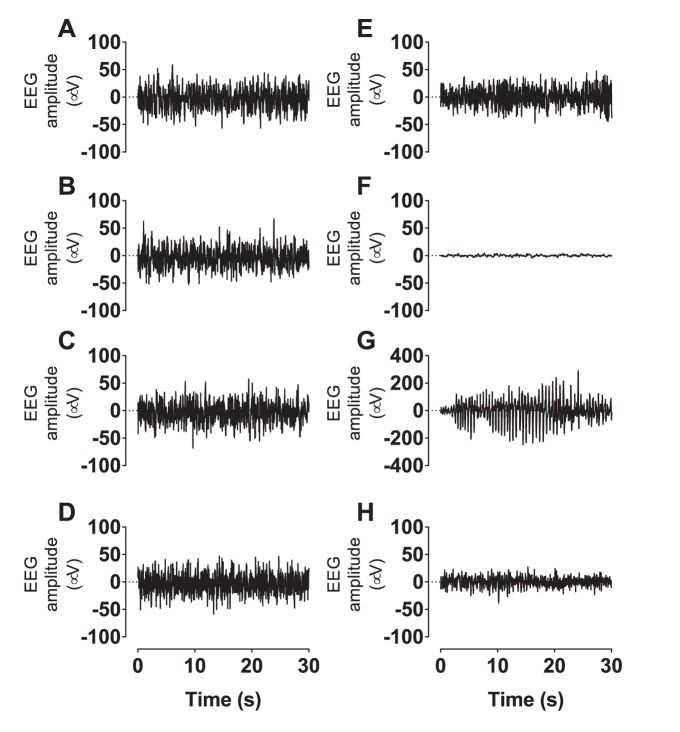
Representative 30(A, E), 30 min after UCO or sham UCO showing marked EEG suppression after occlusion (B, F), during the seizure period, showing an example of an evolving seizure (C, G), and during day 3 of recovery (D, H). Sham UCO example (A–D); UCO example (E–H). Data are continuous EEG recordings saved at 64 Hz.

EEG power was suppressed in the UCO−SE group throughout the recovery period (Figures 2–4, p<0.01 vs. sham occlusion). In contrast, although EEG power in the UCO+SE group was initially suppressed for +1–4 h (p<0.05 vs sham occlusion), power was increased after the onset of status epilepticus (from 6 to 26 h, p<0.05 vs UCO−SE), and then progressively fell to below sham occlusion values from +32 h (p<0.05) and below values in the UCO−SE group from 59 to 72 h (p<0.05). Spectral edge frequency was suppressed in the UCO−SE group until +22 h (p<0.05 vs. sham occlusion), and until +39 h in the UCO+SE group (p<0.05 vs. sham occlusion).

Cortical impedance resolved to sham occlusion values within 1 h after UCO (p<0.05, Figure 2). In the UCO−SE group, impedance remained at sham occlusion values. In contrast, the UCO+SE group showed a secondary rise in impedance from 3.1 (4.7) hours, that peaked at 19.3 (3.2) hours and on average resolved by +29 h (p<0.05 vs. UCO−SE and sham UCO). Seizures began in the UCO−SE group after 25.0 (16.2) hours, compared to 5.8 (6.8) hours in the UCO+SE group (p = 0.07, Mann-Whitney U test). In the UCO+SE group seizure activity was maximal at 14.3 (3.9) hours.

### Histopathology

**Figure 5:**
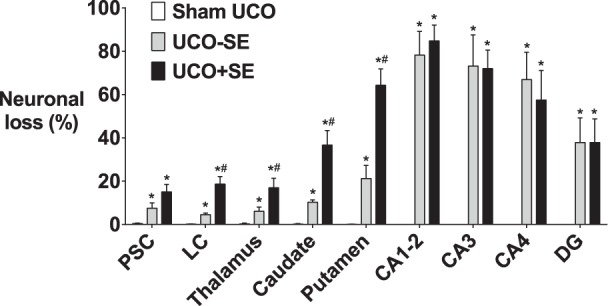
Neuronal loss as estimated by acid-fuchsin thionine staining. UCO groups split according to the presence of status epilepticus (black bars) or no status epilepticus (grey bars). PSC: parasagittal cortex; LC: lateral cortex; CA: cornu ammonis of hippocampus; DG: dentate gyrus. *p<0.05 compared to sham occlusion. #p<0.05 compared to UCO non-status epilepticus group. Data are mean±SEM.

**Figure 6:**
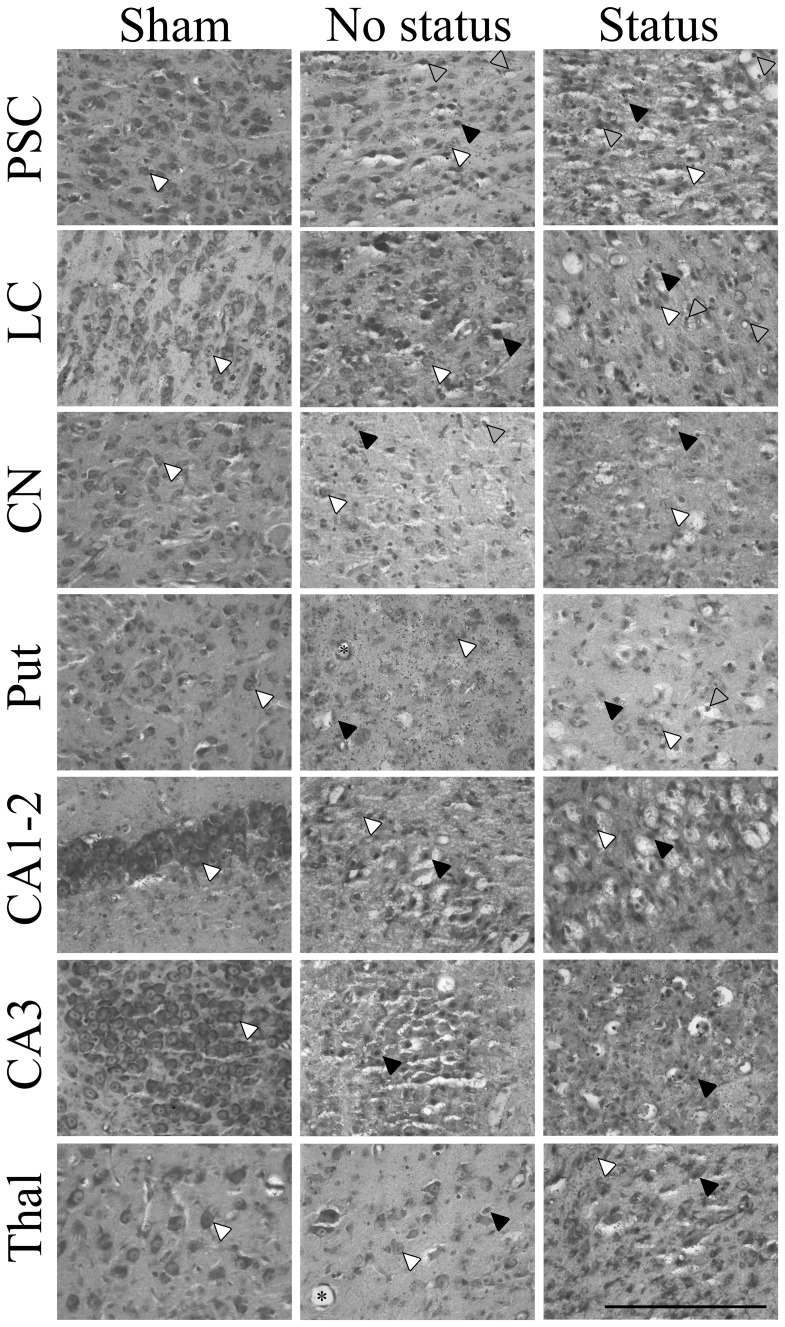
Representative photomicrographs of acid-fuchsin thionine staining. White arrows show examples of healthy thionine stained neurons. Black arrows show examples of dead acid-fuchsin stained neurons. Grey arrows show examples of neurons with condensed nuclei, vacuolated cytoplasm, but not acid-fuchsin positive and thus not counted as dead-neurons. * examples of blood vessels. PSC: parasagittal cortex; LC: lateral cortex; CA: cornu ammonis of hippocampus. ×40 magnification. Scale bar = 200 µm.

No neurons showing ischemic cell change were seen in the hippocampus after sham occlusion, and <1 cell per field with ischemic cell change was seen in the parasagittal cortex, lateral cortex, caudate nucleus, putamen, and thalamus (Figures 5 & 6). The UCO−SE and UCO+SE groups showed relatively mild selective neuronal loss in the parasagittal cortex and lateral cortex (p<0.05 *vs.* sham occlusion), with moderate to severe neuronal loss in the caudate nucleus, putamen and the thalamus (p<0.05 *vs.* sham occlusion) and severe neuronal loss in the cornu ammonis regions of the hippocampus (p<0.05 *vs.* sham occlusion). The presence of status epilepticus was associated with greater neuronal loss in the lateral cortex, basal ganglia and thalamus (p<0.05 *vs.* UCO−SE), but not the hippocampal regions.

## Discussion

The present study demonstrates that in 0.85 gestation fetal sheep prolonged UCO continued until profound hypotension developed was associated with high mortality, and in survivors there was a pattern of predominantly subcortical neuronal loss, with relative sparing of the cortex. This pattern is consistent with the clinical association of severe sentinel events in term infants, such as severe cord prolapse and placental abruption, with a basal ganglia predominant pattern on MRI [2], [3], [4], [5]. Further, UCO was associated with initial EEG suppression followed by delayed onset of evolving seizures, highly consistent with both clinical experience [31], [32] and observations after cerebral ischemia or hypoxia-ischemia in term-equivalent large animals [12], [33], [34]. Approximately half of the surviving fetuses developed periods of status epilepticus, which was associated with greater duration of UCO, a transient secondary increase in cortical impedance (a measure of cytotoxic edema) and subsequently, greater neural injury.

Approximately one third of fetuses in the present study died either immediately after UCO or during recovery, similarly to our previous report [20]. Previous studies of such a prolonged duration of UCO in fetal sheep at the same gestational age as the present study have reported relatively short periods of recovery, likely because of this high mortality [20], [35], [36]. This is also broadly consistent with the typical clinical experience of 30 to 40% mortality after moderate to severe HIE [9], [37], [38]. In addition to cardiac-related mortality, 5 ewes went into labor after UCO and had to be euthanized. Hypothalamic-pituitary-adrenal activation is associated with asphyxia and is a well-established trigger of labor in sheep [39].

Survivors of UCO showed consistently normal to mildly increased arterial blood pressure throughout recovery, but normal fetal heart rate, with reduced femoral blood flow and conductance. This combination of findings strongly implies that cardiac contractility was impaired, with MAP supported by peripheral vasoconstriction. This is consistent with previous findings of increased plasma troponin T levels after 15 min of complete UCO [36], [40], and reversible sub-endocardial injury after prolonged brief repeated UCO in term-equivalent fetal sheep [41]. It is interesting to note that despite this, fetuses were able to maintain spontaneous peripheral vasoconstriction for at least 3 days after this very severe insult. In principle, it may have been possible to improve survival by inotrope infusion, however, in practice we have previously found that infusion of the inotropic agent dopamine delayed but did not prevent secondary hypotension or death in this paradigm [20].

Histologically, prolonged UCO in the present study was associated with predominantly subcortical injury, with severe neuronal loss in the hippocampus, moderate to severe loss in the lentiform nuclei, and relatively mild-moderate injury in the cortex and thalamus. The equivalent pattern seen on MRI in term human neonates with moderate to severe HIE is most often associated with peripartum sentinel events such as cord prolapse and placental abruption [2]. Consistent with the present study, term neonates with acute near total asphyxia (i.e. sentinel events) were more likely to have a longer antenatal duration of bradycardia, lower 5 min Apgar, increased requirement for resuscitation and adrenaline, earlier development of seizures, and more severe adverse outcomes than neonates exposed to prolonged partial asphyxia [42].

Electrophysiologically, UCO was associated with marked initial EEG suppression (as shown in Figures 3 and 4). Further, carotid blood flow and conductance were significant reduced for at least 3 days after occlusion in the present study. Similarly, in acutely exteriorized fetal sheep at a slightly older gestation (134–138 days) 14.5 min of UCO was associated with EEG suppression and reduced cerebral glucose metabolism 4 hours later on positron emission tomography [35]. There is evidence that after UCO the reduction in EEG activity and blood flow in the first few hours reflects, at least in part, an actively mediated reduction in cerebral metabolism [43].

This early suppression was followed by delayed onset of seizures in all fetuses. It remains controversial whether seizures in infants with HIE cause new injury or are a sign of evolving injury [31], [44]. In well oxygenated adult rats induced seizures are associated with neuronal necrosis, particularly in the neocortex and the thalamus [45]. In the piglet, seizures after HI are consistently associated with greater injury, including more abnormal brain metabolites and lower cortical apparent diffusion coefficient on magnetic resonance imaging [33], consistent with the present finding of greater brain swelling. We have previously shown in fetal sheep at the same gestation that after UCO seizures up to ∼3.5 min were associated with normal tissue oxygenation, but when seizures persisted longer than this, moderate but stable deoxygenation occurred [24], suggesting that only prolonged seizures impair oxygenation.

Clinically, there is a close association after perinatal asphyxia between the amount of electrographic seizure activity and subsequent mortality and morbidity [46]. Status epilepticus in particular is associated with a high risk of severe neurologic disability and post-neonatal epilepsy [47]. Recent studies have confirmed that status epilepticus is associated with brain injury even in neonates cooled for HIE [31]. Miller and colleagues found that greater seizure severity was associated with increased lactate/choline and reduced n-acetyl-aspartate/choline ratios on magnetic resonance (MR) spectroscopy after adjustment for abnormalities on MR imaging and resuscitation at birth, confirming that seizures are indeed associated with anerobic metabolism [48]. Nevertheless, that study was not able to define the temporal relationship between the onset or duration of seizures and MR imaging and so, at least in part, this association could be a consequence of evolving injury.

In the present study, status epilepticus was strongly associated with greater neural injury, and with a delayed increase in cortical impedance, a marker of cell swelling [26]. This pattern is highly similar to that seen after 30 minutes of reversible carotid artery occlusion in 0.85 gestation fetal sheep. Critically we have shown that therapeutic hypothermia started before the onset of this secondary deterioration can improve neural outcomes, as previously reviewed [49]. Thus, this pattern strongly infers that UCO triggered evolving neural injury, and raises the possibility that status epilepticus may have exacerbated cortical injury. However, the fetuses that developed status epilepticus were able to survive a greater duration of UCO, and had more severe terminal hypotension, suggesting that more severe primary asphyxia injury underlies the risk of subsequent status. This variation in the timing of onset of terminal hypotension between fetuses must reflect cardiac tolerance to profound anoxia, most likely mediated by differences in cardiac glycogen stores [50]. Thus, it remains unclear whether the UCO+SE group exhibited more severe neural damage as a result of greater initial injury, or as a result of the status epilepticus per se.

It is interesting to contrast the present findings with the effects of 30 min of cerebral ischemia at the same gestation [26]. Cerebral ischemia was associated with more severe cortical injury but only moderate injury of the basal ganglia, in a watershed pattern [51]. Both insults were associated with similar, prolonged suppression of background EEG activity, but spectral edge frequency recovered more slowly after cerebral ischemia than in the present study (Figure 2) [52], consistent with more severe histological cortical injury. After cerebral ischemia, seizures began at 8 hours, with secondary swelling seen from a mean of 7 hours and peaking after 28 to 36 hours [11], [26], [53]. In contrast, in the present study after 17 min of UCO in fetuses developing status epilepticus, seizures began at a median of 6 hours and the secondary increase in impedance was seen markedly earlier, starting at ∼3 hours and reaching a peak after 22 hours. Thus, importantly, these data suggest that secondary neural injury appears to have evolved more rapidly after UCO than isolated forebrain ischemia despite less severe cortical injury.

This difference may reflect a more severe net reduction in delivery of oxygen and nutrients to the brain due to combined hypoperfusion with profound hypoxia during UCO compared with hypoperfusion but normal blood gases during carotid artery occlusion. Alternatively, given the more severe basal ganglia injury in the present study than seen after forebrain ischemia [52], and the known differential regional injury after repeated insults [54], we speculate that it could in part reflect differences in the rate of evolution of injury between regions. Regardless, this finding raises the possibility that UCO may be associated with a relatively shorter window of opportunity for treatment than predicted by studies of ischemia [49]. A potential limitation of the present study is that neuronal loss was assessed after 3 days recovery, and there may be further long-term cell loss [55]; nevertheless severe injury was already present in the basal ganglia and hippocampal regions even at this time.

In conclusion, the present study demonstrates that in fetal sheep at 0.85 of gestation prolonged UCO was associated with high mortality, a high incidence of status epilepticus in survivors, and subsequent severe sub-cortical neuronal loss and mild-moderate cortical injury. The relatively earlier onset of status epilepticus with secondary cortical swelling than previously reported after pure forebrain ischemia in fetal sheep at the same stage of maturity raises the possibility that the window of opportunity for intervention may be shorter than previously appreciated after severe ‘sentinel’ events.
