# Correction: Status Epilepticus after Prolonged Umbilical Cord Occlusion Is Associated with Greater Neural Injury Fetal Sheep at Term-Equivalent

**DOI:** 10.1371/journal.pone.0101908

**Published:** 2014-06-27

**Authors:** The PLOS ONE Staff

The word “in” is missing in the article title and should appear after the word “injury.” The correct title is: Status Epilepticus after Prolonged Umbilical Cord Occlusion Is Associated with Greater Neural Injury in Fetal Sheep at Term-Equivalent.

The correct citation is: Drury PP, Davidson JO, van den Heuij LG, Wassink G, Gunn ER, et al. (2014) Status Epilepticus after Prolonged Umbilical Cord Occlusion Is Associated with Greater Neural Injury in Fetal Sheep at Term-Equivalent. PLoS ONE 9(5): e96530. doi:10.1371/journal.pone.0096530

## References

[1] Drury PP, Davidson JO, van den Heuij LG, Wassink G, Gunn ER, et al (2014) Status Epilepticus after Prolonged Umbilical Cord Occlusion Is Associated with Greater Neural Injury Fetal Sheep at Term-Equivalent. PLoS ONE 9(5): e96530 doi:10.1371/journal.pone.0096530 24797081 10.1371/journal.pone.0096530 PMC4010475

